# Genome-wide analysis of the polyphenol oxidase gene family reveals that MaPPO1 and MaPPO6 are the main contributors to fruit browning in *Musa acuminate*

**DOI:** 10.3389/fpls.2023.1125375

**Published:** 2023-02-14

**Authors:** Fei Qin, Chunhua Hu, Tongxin Dou, Ou Sheng, Qiaosong Yang, Guiming Deng, Weidi He, Huijun Gao, Chunyu Li, Tao Dong, Ganjun Yi, Fangcheng Bi

**Affiliations:** ^1^ Key Laboratory of South Subtropical Fruit Biology and Genetic Resource Utilization (Ministry of Agriculture and Rural Affairs), Guangdong Provincial Key Laboratory of Tropical and Subtropical Fruit Tree Research, Institute of Fruit Tree Research, Guangdong Academy of Agricultural Sciences, Guangzhou, China; ^2^ Guangdong Laboratory for Lingnan Modern Agriculture, Guangzhou, China; ^3^ College of Life Sciences, South China Agricultural University, Guangzhou, China

**Keywords:** *musa acuminate*, PPO gene family, expression pattern, polyphenol oxidase activity, fruit browning

## Abstract

**Introduction:**

Polyphenol oxidases (PPOs), which are widely present in plants, play an important role in the growth, development, and stress responses. They can catalyze the oxidization of polyphenols and result in the browning of damaged or cut fruit, which seriously affects fruit quality and compromises the sale of fruit. In banana (*Musa acuminata*, AAA group), 10 *PPO* genes were determined based on the availability of a high-quality genome sequence, but the role of *PPO* genes in fruit browning remains unclear.

**Methods:**

In this study, we analyzed the physicochemical properties, gene structure, conserved structural domains, and evolutionary relationship of the *PPO* gene family of banana. The expression patterns were analyzed based on omics data and verified by qRT-PCR analysis. Transient expression assay in tobacco leaves was used to identify the subcellular localization of selected MaPPOs, and we analyzed the polyphenol oxidase activity using recombinant MaPPOs and transient expression assay.

**Results and discussion:**

We found that more than two-thirds of the *MaPPO* genes had one intron, and all contained three conserved structural domains of PPO, except *MaPPO4*. Phylogenetic tree analysis revealed that *MaPPO* genes were categorized into five groups. MaPPOs did not cluster with Rosaceae and Solanaceae, indicating distant affinities, and MaPPO6/7/8/9/10 clustered into an individual group. Transcriptome, proteome, and expression analyses showed that MaPPO1 exhibits preferential expression in fruit tissue and is highly expressed at respiratory climacteric during fruit ripening. Other examined *MaPPO* genes were detectable in at least five different tissues. In mature green fruit tissue, *MaPPO1* and *MaPPO6* were the most abundant. Furthermore, MaPPO1 and MaPPO7 localized in chloroplasts, and MaPPO6 was a chloroplast- and Endoplasmic Reticulum (ER)-localized protein, whereas MaPPO10 only localized in the ER. In addition, the enzyme activity *in vivo* and *in vitro* of the selected MaPPO protein showed that MaPPO1 had the highest PPO activity, followed by MaPPO6. These results imply that MaPPO1 and MaPPO6 are the main contributors to banana fruit browning and lay the foundation for the development of banana varieties with low fruit browning.

## Introduction

Polyphenol oxidase (PPO) is a copper-binding enzyme that widely exists in animals, plants, fungi, and bacteria (Mayer, 2006). According to its specific substrate and mechanism of action, it can be divided into three categories: tyrosinases (EC 1.14.18.1), catechol oxidases (EC 1.10.3.1), and laccase (EC 1.10.3.2)(Mishra and Gautam, 2016). Many studies have reported that PPO is induced in response to biotic and abiotic stress in plants, and it has been implicated in several functional processes, including participating in plant defense and the synthesis of plant-specific metabolites (Constabel et al., 1995; Gandía-Herrero and García-Carmona, 2013; Araji et al., 2014; Sullivan, 2015).

The browning reaction of plants is considered to be related to PPO. The oxidation of phenolic substrates by polyphenol oxidase (EC 1.10.3.1) is thought to be the major cause of the brown discoloration of many fruit and vegetables during harvesting, storage, transportation, and processing (Vámos-Vigyázó and Haard, 1981; Queiroz et al., 2008; Moon et al., 2020). Catecholase is mainly distributed in plants and typically catalyzes the oxidation of o-diphenols to o-quinones in the presence of molecular oxygen. Quinones are highly reactive and spontaneously cross-link with amino acids, proteins, and other phenolic compounds to form brown polymers that appear in plant extracts and wounded tissues (Mayer, 2006; Sullivan, 2015), which also cause significant economic impacts, both to primary food producers and the food processing industry (Singh et al., 2018; Dias et al., 2020).

Plant PPO proteins generally contain three conserved regions, namely an N-terminal cTP, a CuA and CuB (tyrosinase) domain, and a C-terminus extension (Tran et al., 2012), which are responsible for thylakoid lumen localization and enzyme activity. PPO proteins are found in many species of terrestrial plants, such as apple (Guardo et al., 2013), strawberry (Jia et al., 2016), potato (Chi et al., 2014), tomato (Newman et al., 1993), banana (Gooding et al., 2001), Populus (He et al., 2021), rice (YanchunYu et al., 2008), wheat (Beecher et al., 2012), barley (Taketa et al., 2010), and eggplant (Jukanti and Bhatt, 2015), as well as in fungi (Li et al., 2011) and bacteria (Gorshkov et al., 2017). However, no homologs of the PPO gene were found in the *Arabidopsis thaliana* genome (Tran et al., 2012).

The distribution and function of PPO proteins differ in different plants (Tran et al., 2012). Most PPO proteins are transported to the thylakoid lumen in the chloroplast (Koussevitzky et al., 2008), and they have also been found in vacuoles, the cytosol, and other organelles (Nakayamaa et al., 2001; Tran and Constabel, 2011), while phenolic compounds are generally confined to the vacuoles (Vaughn and Dnke, 1984). Given the physical separation of PPO enzymes from their substrates, the PPO enzyme–substrate interaction requires the destruction of cell compartmentation by insect, mechanical damage, diseases, or microorganism invasion (Boeckx et al., 2015; Maioli et al., 2020).

Banana (*Musa acuminata*, AAA group) is one of the world’s most important fruit crops and is widely cultivated in tropical countries due to its high nutritional and economic value (Padam et al., 2014). However, enzymatic browning has a serious impact on the development of the banana industry, especially in the process of harvesting and post-harvest, such as during handling, storage, and processing (Gooding et al., 2001). The approach to the prevention of enzymatic browning is divided into physical and chemical methods. Physical methods to regulate enzymatic browning include thermal treatment, prevention of oxygen exposure, use of low temperature, and irradiation (Tinello and Lante, 2018). Chemical methods to inhibit PPO activity include acidification or reduction using antioxidants, chelating agents, or natural extracts (Moon et al., 2020). Most of these methods have negative consequences. Therefore, the most attractive method for preventing food browning is through natural methods (Hamdan et al., 2022). Therefore, the development of new banana varieties with low fruit browning is the best way to tackle this problem through molecular breeding approaches, such as genome editing.

In banana, Gooding et al., 2001 identified four *MaPPO* genes from the Cavendish subgroup and indicated that fruit browning during ripening is due to the release of the pre-existing enzyme through detecting the transcripts of *MaPPO* genes. The detailed sequence information of these four *MaPPOs* is not available, and the main contributors to fruit browning remain obscure. In this study, the *PPO* genes were analyzed genome-wide in *Musa acuminate*, and 10 putative *PPO* genes were identified. The expression patterns of *MaPPOs* were comparatively examined based on transcriptome, proteome, and real-time reverse transcription-PCR (qRT-PCR) data. Moreover, the subcellular localization of the four MaPPOs was identified using transient expression in tobacco leaves. In addition, the PPO activity of the selected MaPPOs was analyzed *in vivo* and *in vitro*. The results shed light on the role of MaPPOs in fruit browning and provide a theoretical basis for creating new varieties with low fruit browning.

## Materials and methods

### Determination of *PPO* genes in *Musa acuminate*

BLAST and HMMER were used to identify *PPO* genes with conserved structures in banana. *Musa acuminate* amino acid sequences (MaPPOs) were extracted from the *M. acuminate* assembly (https://banana-genome-hub.southgreen.fr/). To examine the presence of the conserved domain, a batch search of the sequences for all obtained *MaPPO* genes was performed through the online databases of SMART (http://smart.embl.de/smart/set_mode.cgi?GENOMIC=1) and NCBI CDD (https://www.ncbi.nlm.nih.gov/cdd/). The MWs, PIs, and hydrophilia parameters were evaluated using an online tool on the ExPasy server (https://web.expasy.org/protparam/).

### Phylogenetic analysis of *MaPPO* genes

Protein sequences of PPOs from *Malus domestica* (MdPPO), *Oryza sativa* (OsPPO), *Solanum lycopersicum* (SlPPO), *Solanum tuberosum* (StPPO), *Triticum aestivum* (TaPPO), *Hordeum vulgare* (HvPPO), *Fragaria ananassa* (FaPPO), and *Solanum melongena* (SmPPO) were obtained from the Phytozome database (Goodstein et al., 2012). Multiple sequence alignment of MaPPO proteins was performed using the MUSCLE program in MEGA11 software, and the phylogenetic tree was constructed using the NJ method with the Poisson model, pairwise deletion, and 1000 repeats. The graph of the phylogenetic tree was modified using the online tool Evolview (https://www.evolgenius.info/evolview/).

### Determination of gene and protein structures and motifs

The gene structure, including introns, coding sequences (CDSs), and untranslated regions (UTRs), of banana *MaPPOs* was derived from the banana genome (https://banana-genome-hub.southgreen.fr/node/50/7720969) and displayed using the Gene Structure Display Server (http://gsds.cbi.pku.edu.cn/). MEME (https://meme-suite.org/meme/tools/meme) and the NCBI Conserved Structural Domain Database CDD (https://www.ncbi.nlm.nih.gov/Structure/bwrpsb/bwrpsb.cgi) were used to identify the conserved motifs and conserved structural domains of MaPPO proteins, respectively. The conserved motifs and structures of all PPO proteins in banana were displayed using TBtools (https://github.com/CJ-Chen/TBtools).

### Plant material and treatments

Mature green banana fruit (*Musa acuminata* AAA group, Cavendish subgroup) was obtained from a local market in Guangdong, China. Ethephon-induced ripening, 1-MCP-delayed ripening, and natural ripening of the fruit were evaluated as described previously (Li et al., 2020). Each treatment contained three biological replicates and was stored at 22°C with about 90% relative humidity until fully ripe. The fruit samples from various development stages were collected from local banana plantations in Guangzhou, China. The sampling time point was determined by the number of days after flowering. The fruit samples at each time point were collected and quickly frozen in liquid nitrogen and then stored at −80°C until utilization.

### RNA extraction and qRT-PCR analysis

The fine powder of each sample ground in liquid nitrogen was used to isolate total RNA, as described in a previous report (Asif et al., 2000). The elimination of any potentially contaminated gDNA and reverse transcription of cDNA were performed using a reverse transcription kit (TaKaRa, Dalian, China) according to the manufacturer’s protocol. qRT-PCR and data analysis were conducted as per our previously reported method (Li et al., 2020), and *MaCAC* (clathrin adaptor complex medium subunit, HQ853240) was used as a reference gene (Chen et al., 2011). The primers with high amplification efficiency (90–110%) were designed using Beacon Designer 7, and these are listed in **Supplementary File 2**. The qRT-PCR assay was conducted using the Applied Biosystems Q5 Real-Time PCR System (ThermoFisher, USA) using TB Green^®^ Premix kit (Tli RNaseH Plus, TAKARA, Dalian, China).

### Analysis of the expression patterns of *MaPPOs* using transcriptomic data

The expression data of *MaPPO* genes during fruit ripening were retrieved from our previous transcriptomic data on fruit ripening (Li et al., 2020), and proteomic analysis of the same samples was performed at the same time. The protein expression level of MaPPOs was obtained using proteome data. For analysis of expression patterns during fruit development, banana fruit was collected 15, 30, 45, 60, and 75 DAF (days after flower), and total RNA was extracted for sequencing analysis by Nuoji Biotechnology Company (China). The relative expression of *MaPPO* genes or proteins was displayed as a heat map drawn with TBtools (Chen et al., 2020). The raw reads of the transcriptome were deposited in the National Center for Biotechnology Information Sequence Reads Archive (SRA) under accession number PRJNA598018.

### Subcellular localization analysis

The CDSs of each *MaPPO* without the stop codon were amplified by PCR from cDNA and introduced into the pCambia1300-GFP vector (modified from pCambia1300). The expression of GFP and GFP-infused MaPPOs was driven by the cauliflower mosaic virus (CaMV)35S promoter. The fusion constructs and the control GFP vector were transiently expressed in tobacco leaf cells using the *Agrobacterium*-mediated method. The assay was performed as described previously (Sparkes et al., 2006). The ER tracker (Mravec et al., 2009) was co-expressed to indicate the localization of ER. GFP signals were examined with a fluorescence microscope (Zeiss LSM 710).

### Prokaryotic expression and determination of PPO activity

The CDSs of *MaPPO* genes were introduced into the *E. coli* expression vector pCZN1. Protein expression was performed under the following conditions: an initial culture (OD600 = 0.5), followed by addition of 0.2 mM IPTG, and incubation at 15°C overnight. Cell lysates were examined by immunity blots using an anti-His tag antibody (Tiangen, Beijing, China). The recombinant MaPPO proteins were purified on Ni-NTA His-Bind resin (Novagen, USA), and the protein concentration was assayed using an Easy Protein Quantitative Kit (Trans, Beijing, China). PPO activity was conducted as described in the previous report using a polyphenol oxidase activity assay kit (Beijing Solarbio Science & Technology Co., Ltd., Beijing)(https://doi.org/10.1007/s11947-018-2232-0). PPO activity was calculated based on the amount of recombinant protein or the fresh weight of the tissue sample in the reaction system.

### Transient overexpression of *MaPPOs* in banana fruit

The CDS of *MaPPO* gene were subcloned into pCMABIA1300 vector. The *A. tumefaciens* strain EHA105 including the constructed plasmids or control vector was injected into the mature-green banana fruits through the distal end using a syringe. The middle three hands from each banana bunch were used the experiment. At least eight fruit fingers were used for each treatment, and four ml *A. tumefaciens* was delivered into each fruit finger. One day after bacterium infiltration, injected fruits were dipped into ethephon solution (1000 times dilution from 40% ethephon) for 1 min, and stored at 22°C with 90% relative humidity for 5 d. Samples were harvested on Day 3 for examination of gene expression and PPO activity.

## Results

### Determination of polyphenol oxidase genes in Cavendish banana

Through a genome-wide search in the genome database of *M. acuminate* (https://banana-genome-hub.southgreen.fr/tripal_megasearch), a total of 10 full-length *MaPPOs* were identified. To identify the candidate coding sequences (CDSs) of the 10 *MaPPOs*, the coding regions were amplified with PCR using a cDNA template. The final *MaPPOs* were referred to as *MaPPO1-MaPPO10* according to their order on the chromosomes. The concrete sequences of each *PPO* gene in cultivated banana (*Musa acuminata* AAA group, Cavendish subgroup) were determined by PCR amplification from cDNA and sequencing, and the detailed sequences are listed in **Supplementary File 1**. The characterization of these *MaPPOs* is presented in **Table 1**. The deduced MaPPO proteins had amino acid numbers from 238 to 595, molecular weights (MWs) from 25.76 to 67.57 kDa, hydrophilia parameters from -0.572 to -0.157, and isoelectric points (PIs) from 5.87 to 9.09.

**Table 1:** Charcterization of *MaPPOs*.

Name	Gene ID	Organism	Version	AA	CDS	gDNA	MW	PI	GRAVY
MaPPO1	Ma06_t31080.1	DH-Pahang	2	586	1758	2839	64.65	6.17	-0.339
MaPPO2	Ma07_t03540.1	DH-Pahang	2	588	1764	1935	65.54	6.97	-0.395
MaPPO3	Ma07_t03650.1	DH-Pahang	2	536	1611	10192	60.80	7.25	-0.562
MaPPO4	Ma08_t00340.1	DH-Pahang	2	238	717	1025	25.76	9.09	-0.157
MaPPO5	Ma08_t34740.1	DH-Pahang	2	590	1773	1952	65.90	6.53	-0.503
MaPPO6	Ma08_t09150.1	DH-Pahang	2	590	1773	2229	67.12	6.77	-0.545
MaPPO7	Macma4_08_g09060.1	DH-Pahang	4	595	1788	/	67.57	6.77	-0.497
MaPPO8	Ma08_t09170.1	DH-Pahang	2	592	1779	2004	67.30	7.02	-0.548
MaPPO9	Macma4_08_g09080.1	DH-Pahang	4	588	1767	/	66.97	7.25	-0.572
MaPPO10	Ma10_t20510.1	DH-Pahang	2	577	1734	3788	66.41	5.87	-0.525

AA, amino acid; CDS, coding sequence; pI, theoretical isoelectric point; MW, molecular weight; GRAVYY, grand average of hydropathicity;/, non determined.

### Phylogenetic analysis of MaPPOs and PPOs from other plant species

**Figure 1:**
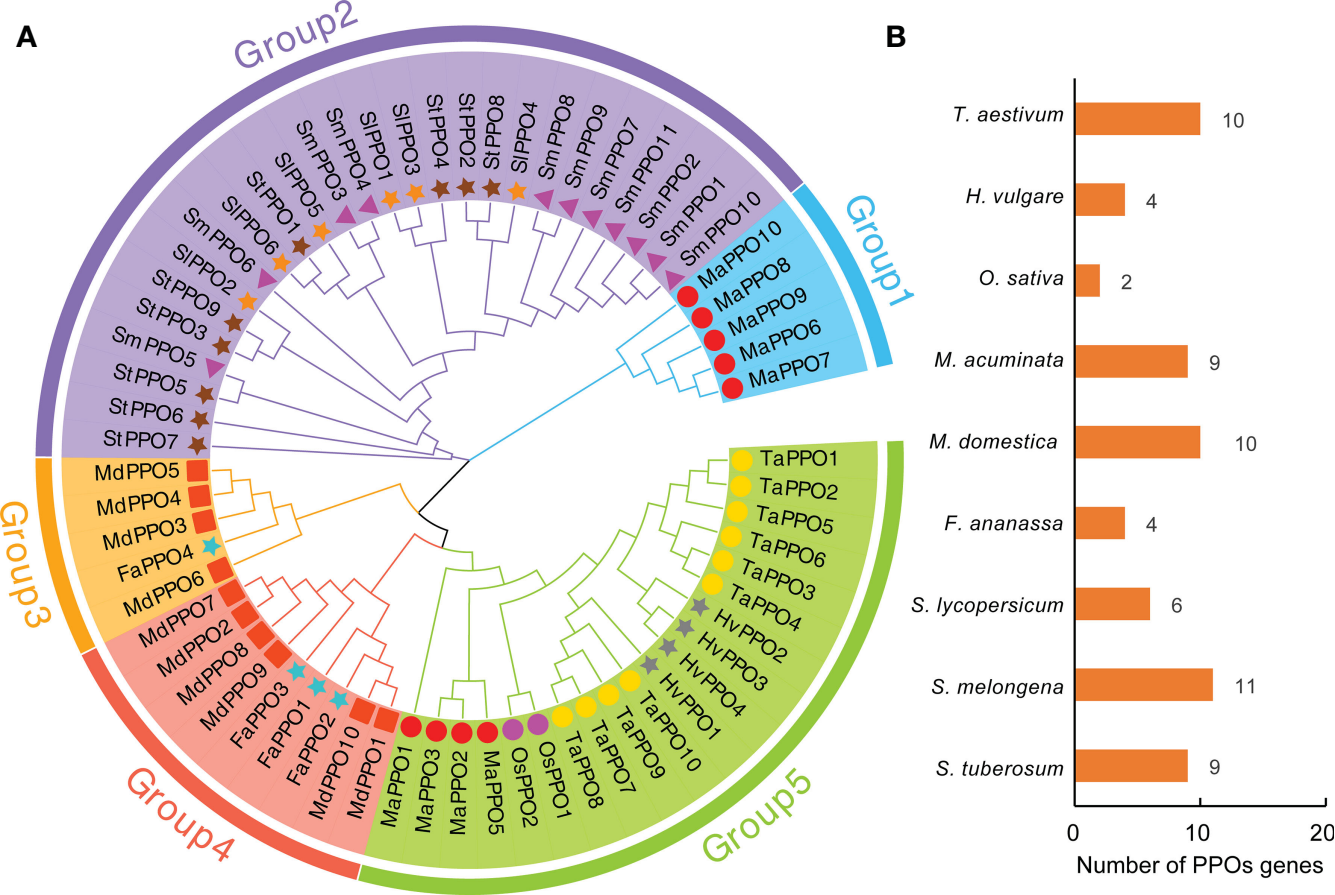
Phylogenetic analysis of PPO proteins in nine plant species. **(A)** Phylogenetic relationship of PPO proteins from *Musa acuminate* (MaPPO), *Malus domestica* (MdPPO), *Oryza sativa* (OsPPO), *Solanum lycopersicum* (SlPPO), *Solanum tuberosum* (StPPO), *Triticum aestivum* (TaPPO), *Hordeum vulgare* (HvPPO), *Fragaria ananassa* (FaPPO), and *Solanum melongena* (SmPPO). The phylogenetic tree was built using the neighbor-joining (NJ) method implemented in MEGA11 with 1000 bootstrap replicates. The PPOs identified from different species are indicated by different shapes and colors in front of the node. **(B)** Presentation of PPO protein numbers across nine plant species.

To investigate the evolutionary relationship of the *PPO* gene family, an unrooted neighbor-joining (NJ) tree was constructed with 65 PPO proteins from wheat, barley, rice, banana, apple, strawberry, tomato, eggplant, and potato. The homologs of MaPPO4 were not included in the analysis because the conserved domain of MaPPOs was incomplete. As shown in **Figure 1**, the phylogenetic tree was characterized by five subgroups. The largest number of PPOs was observed in Group 2, with 26 PPOs, followed by Groups 5 (20) and 4 (9). Remarkably, MaPPOs were only distributed in Groups 1 and 5, and Group 1 had only 5 PPO proteins from banana and none from other examined plant species, suggesting that these PPOs might have been obtained in banana after their divergence or loss in other plant species. The remaining four PPO proteins in banana were concentrated in Group 5 and clustered in the same branch as other PPOs from monocots (wheat, barley, rice), indicating that they might have evolved from common ancestors. Interestingly, each group included PPO proteins from either monocots or dicots, and the PPO proteins from dicots were clustered into three individual groups (groups 2, 3, and 4), indicating that PPOs from monocots and dicots evolved independently from different lineages.

### Gene structure and motif analysis of *MaPPO* genes

**Figure 2:**
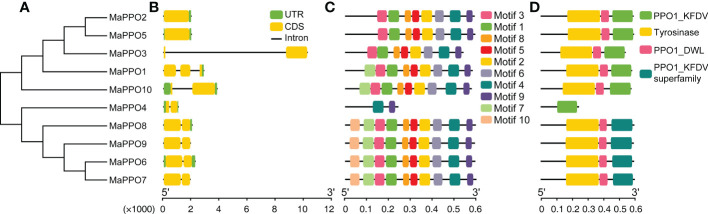
Phylogenetic, gene structure, motif, and conserved protein structure analyses of MaPPOs. **(A)** Phylogenetic relationship of *MaPPOs*. **(B)** Gene structure analysis using the genome sequences of *MaPPO* genes. Green boxes indicate UTRs; yellow boxes indicate exons; black lines indicate introns. **(C)** Motif presentation of the MaPPO members. **(D)** Conserved domain of MaPPOs.

To gain more insights from the evolutionary relationship within the banana *MaPPO* gene family, a phylogenetic tree of 10 PPO proteins from banana was constructed using MEGA 11 using the NJ method with 1000 bootstrap replicates (**Figure 2A**). The motif architectures, conserved protein structures, and gene structures of 10 *MaPPOs* were examined within the phylogenetic context and visualized using TBtools. Seven out of 10 *MaPPOs* had one intron; one *MaPPO* had two introns, and two *MaPPOs* had no introns (**Figure 2B**). Interestingly, two *MaPPOs* (*MaPPO3/7*) had no untranslated regions (UTR) structure (**Figure 2B**). The phylogenetic tree of the *MaPPO* genes contained two subgroups. Generally, a gene within the same group had a similar and conserved structure in terms of exon number and intron length; for instance, all members in Group 2 had two exons and one intron. Based on searching the CDD and SMART databases, we identified three conserved domains (PPO1_KFDV, tyrosinase, and PPO1_DWL) in the MaPPO proteins and found them in all MaPPOs, except in MaPPO4, indicating their conserved biological functions (**Figure 2D**). Moreover, we identified 10 conserved motifs in the PPO protein sequence using MEME (**Figure 2C**), and five motifs (1, 2, 3, 5, and 8), one motif (6), and two motifs (4 and 9) were related to the N-terminal tyrosinase, intermediate PPO1_DWL, and C-terminal PPO1_KFDV domains, respectively. In addition, among most MaPPOs, two novel motifs (10 and 7) were identified at the N-terminal of the protein sequences.

### Expression profiles of *MaPPO* genes in various tissues of banana

**Figure 3:**
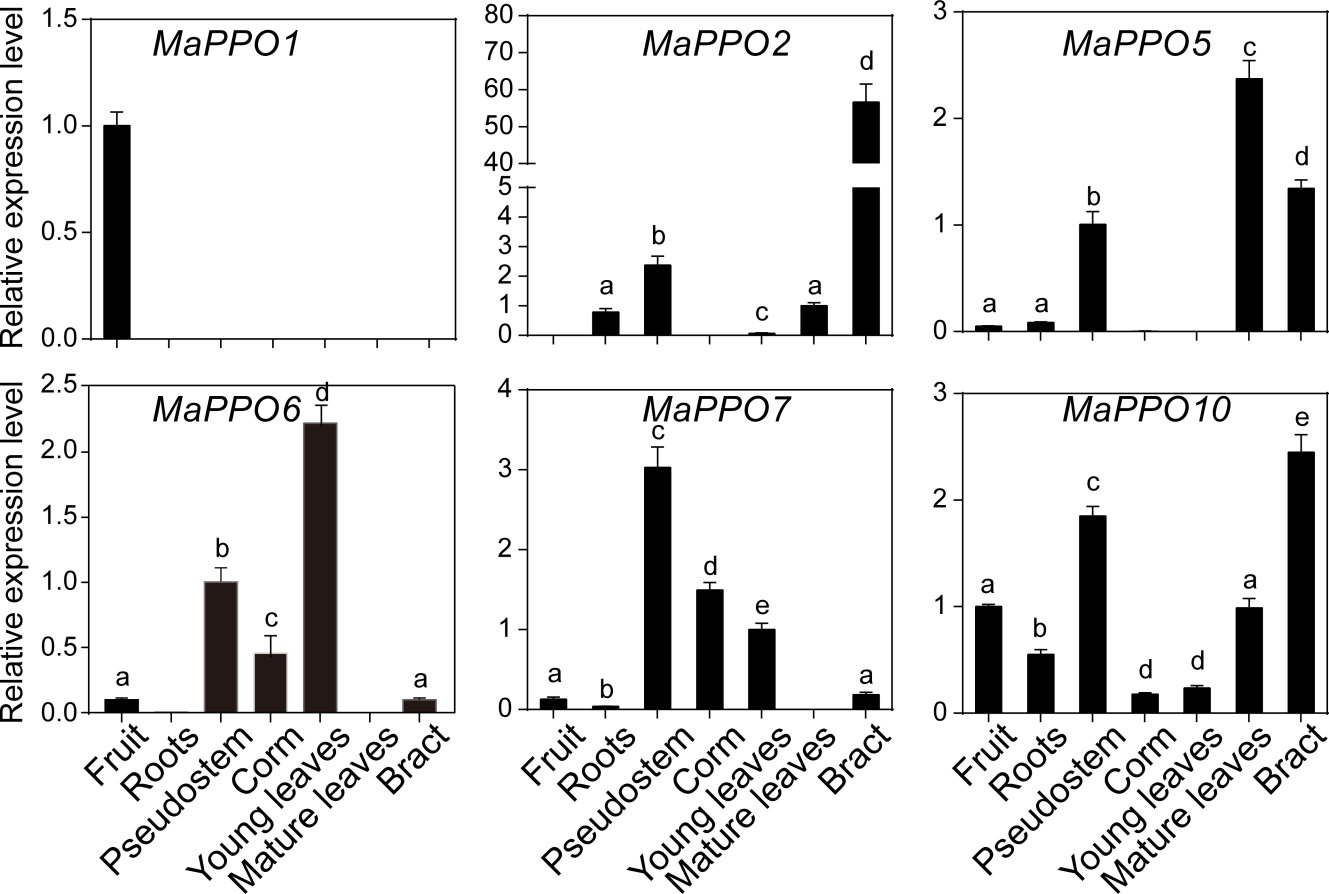
Expression profiles of *MaPPOs* in various tissues of *Musa acuminate.* The transcript level in one tissue was arbitrarily set to 1. Error bars represent the standard deviations of the mean value from three biological and three technical replicates. Different letters indicate significant differences (P < 0.05) examined by one-way analysis of variance (ANOVA).

**Figure 4:**
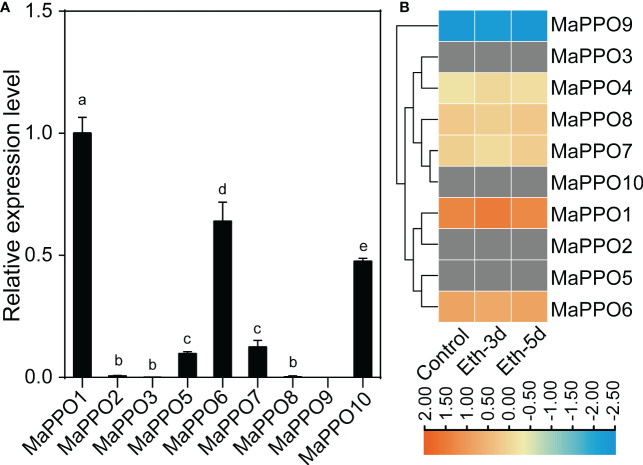
Comparison of expression levels of polyphenol oxidase in fruit. **(A)** qRT-PCR analysis of the expression level of *MaPPOs* in mature green fruit. **(B)** Expression level of MaPPO proteins in response to ethephon. The heatmap was generated using proteome data. The color bar indicates the range of maximum and minimum values of relative expression in the heatmap. The gray color indicates that the protein is undetectable. Different letters indicate significant differences (P < 0.05) examined by one-way ANOVA.

To investigate the roles of *MaPPOs*, we examined the expression levels of nine *MaPPO* genes in roots, pseudostems, corms, young leaves, mature leaves, bracts, and mature green fruit of banana using qRT-PCR (**Figure 3**). Three *MaPPO* genes (*MaPPO3/8/9*) were undetectable in all examined tissues due to their low transcript abundance (data not shown). *MaPPO1* was preferentially expressed in mature green fruit, and *MaPPO2* and *MaPPO10* were predominantly expressed in bract tissue. *MaPPO6* and *MaPPO7* had similar expression patterns and were highly expressed in young leaves, whereas *MaPPO5* was highly expressed in mature leaves (**Figure 3**). Differential expression of various *MaPPOs* could imply their different functions in various plant tissues. To further understand the role of *MaPPO* genes in fruit tissue, the transcript levels of all *MaPPOs* were compared. The data revealed that *MaPPO1* had the highest expression in fruit, followed by *MaPPO6*, *MaPPO10*, *MaPPO7*, and *MaPPO5* (**Figure 4A**). Moreover, *MaPPO1* and *MaPPO6* had the highest protein expression levels in the proteome data of banana fruit (**Figure 4B**), suggesting that they might play an important role in fruit tissues.

### Expression analysis of *MaPPO* genes during fruit development and ripening

**Figure 5:**
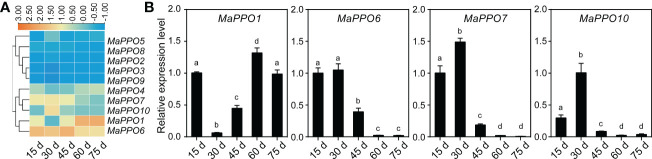
Expression profile of *MaPPO* genes under different fruit developmental stages. **(A)** Heatmaps indicating the expression levels of *MaPPO* genes based on transcriptome data. **(B)** Confirmation of the expression levels of *MaPPO* genes at different developmental stages. Different letters indicate significant differences (P < 0.05) examined by one-way ANOVA.

**Figure 6:**
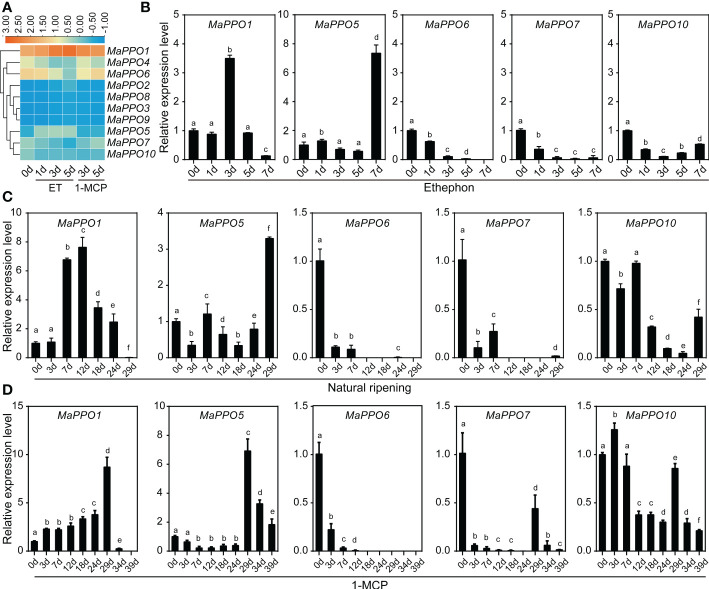
Expression profile of *MaPPO* genes in banana under different treatments. **(A)** Heatmaps showing the expression levels of 10 *MaPPO* genes in response to ethephon and 1-MCP. **(B–D)** Determination of the expression level of *MaPPO* genes under ethephon treatment **(B)**, natural conditions **(C)**, and 1-MCP treatment **(D)**. Different letters indicate significant differences (P < 0.05) examined by one-way ANOVA.

To determine the role of *MaPPOs* in fruit development and ripening, the transcriptomic data of the MaPPOs were analyzed. The expression of four genes (*MaPPO1/6/7/10*) had a relatively high transcript abundance and varied with stage (**Figure 5**), whereas *MaPPO2/3/5/8/9* were almost not detected throughout fruit development (data not shown). qRT-PCR analysis indicated that the expression patterns of these *MaPPOs* were similar to those observed in the transcriptomic analysis (**Figure 5**). At the early developmental stage of fruit, *MaPPO6/7/10* exhibited a high expression level, which decreased gradually to a very low level at 75 days after flowering (DAF) (**Figure 5**). The *MaPPO1* transcript was present at a relatively high level at 15 DAF, which decreased at 30 DAF and then increased to the highest expression level at later stages of development (**Figure 5**). During the postharvest ripening of banana fruit, transcriptomic data showed that *MaPPO1/4/5/6/7/10* were detectable after ethephon treatment, and *MaPPO1* presented the highest expression level among them, whereas the other four *MaPPOs* were almost undetectable (**Figure 6A**). To confirm the reliability of *PPO* gene expression in the transcriptome, the expression of the *MaPPO* genes was examined using qRT-PCR. *MaPPO4* was not included in the following experiments due to its missing PPO-KFDV and PPO_DWL domains. A similar expression pattern of the *MaPPO* gene to that in the transcriptome data was observed during the ripening process (**Figure 6B**). In response to ethephon treatment, the expression of *MaPPO1* increased to the highest level at day 3 and then decreased gradually; the other four detectable MaPPO genes showed relatively low abundance. *MaPPO6* and *MaPPO7* exhibited gradually decreased expression patterns. *MaPPO5* presented a relatively higher expression in the fruit senescence stage, whereas *MaPPO10* presented an expression trend of decreasing first and then increasing throughout the ripening process. Under natural ripening and 1-Methylcyclopropene (1-MCP) treatment, a trend similar to that of ethephon treatment was observed, and the expression of each *MaPPO* was delayed with the postponement of the fruit ripening process (**Figure 6**). Interestingly, *MaPPO1* and *MaPPO6* exhibited higher transcript levels both in the early fruit development stage (**Figure 5A**, 15 d) and the mature green stage (**Figure 4A**; **Figure 6A**, 0 d) and had higher protein expression levels in the mature green fruit stage (**Figure 4B**, control), compared to other *MaPPO* genes.

### Subcellular localization of MaPPOs

**Figure 7:**
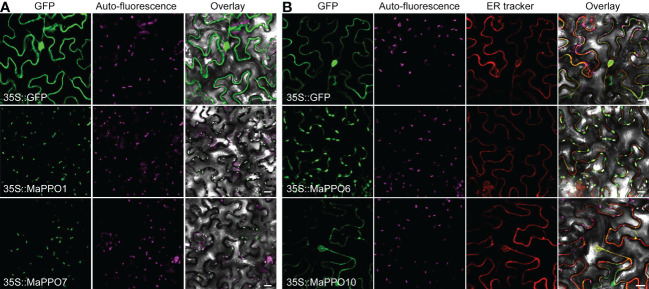
Subcellular localization of selected MaPPO proteins in transient tobacco leaves. **(A)** Subcellular localization of MaPPO1 and MaPPO7; **(B)** Subcellular localization MaPPO6 and MaPPO10. The GV3101 strains containing corresponding GFP-fused plasmids or endoplasmic reticulum (ER)-mCherry were infiltrated into *Nicotiana benthamiana* leaves. Fluorescence signals were examined using a confocal laser scanning microscope. Bar = 50 μm.

To further investigate the role of *MaPPOs* in fruit development and ripening, four relatively high-expression *MaPPO* genes were selected to examine subcellular localization. The CDS of each *PPO* gene was infused to the C-terminus of GFP (**Figure 7**). These GFP chimeras were transiently expressed in tomato leaf cells, and the localization of the expressed proteins was examined using confocal laser microscopy 48 h after *Agrobacterium* infection. The fluorescence signal of 35S::GFP was observed in the nucleus and cytoplasm (**Figure 7**), whereas the signal of MaPPO1::GFP and MaPPO7::GFP overlapped with the auto-fluorescence of chloroplasts (**Figure 7A**), suggesting that MaPPO1 and MaPPO7 are chloroplast-localized proteins. The fluorescence signal of MaPPO6::GFP was observed both in chloroplasts and ER, and the signal of MaPPO10::GFP was co-localized with that of the endoplasmic reticulum (ER) marker, indicating that MaPPO6 was a chloroplast- and ER-localized protein and MaPPO10 was an ER-localized protein (**Figure 7**).

### Polyphenol oxidase activity of MaPPO proteins

**Figure 8:**
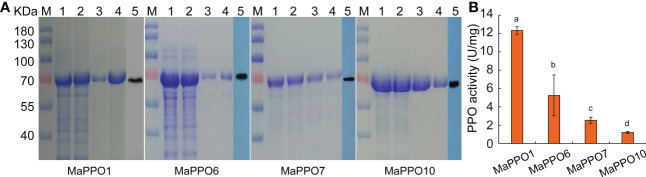
Prokaryotic expression, purification, and enzyme activity analysis of selected MaPPO proteins. **(A)** SDS-PAGE of MaPPO1, MaPPO6, MaPPO7, and MaPPO10 at different purification stages. M, Molecular weight marker; 1, Bacterial lysate, 2, Total soluble protein fraction; 3, 4, Purified His-fusion; 5, Immunodetection of His-PPO using an anti-His antibody. **(B)** Polyphenol oxidase activity of purified recombinant MaPPO proteins. Different letters indicate significant differences (P < 0.05) examined by one-way ANOVA.

**Figure 9:**
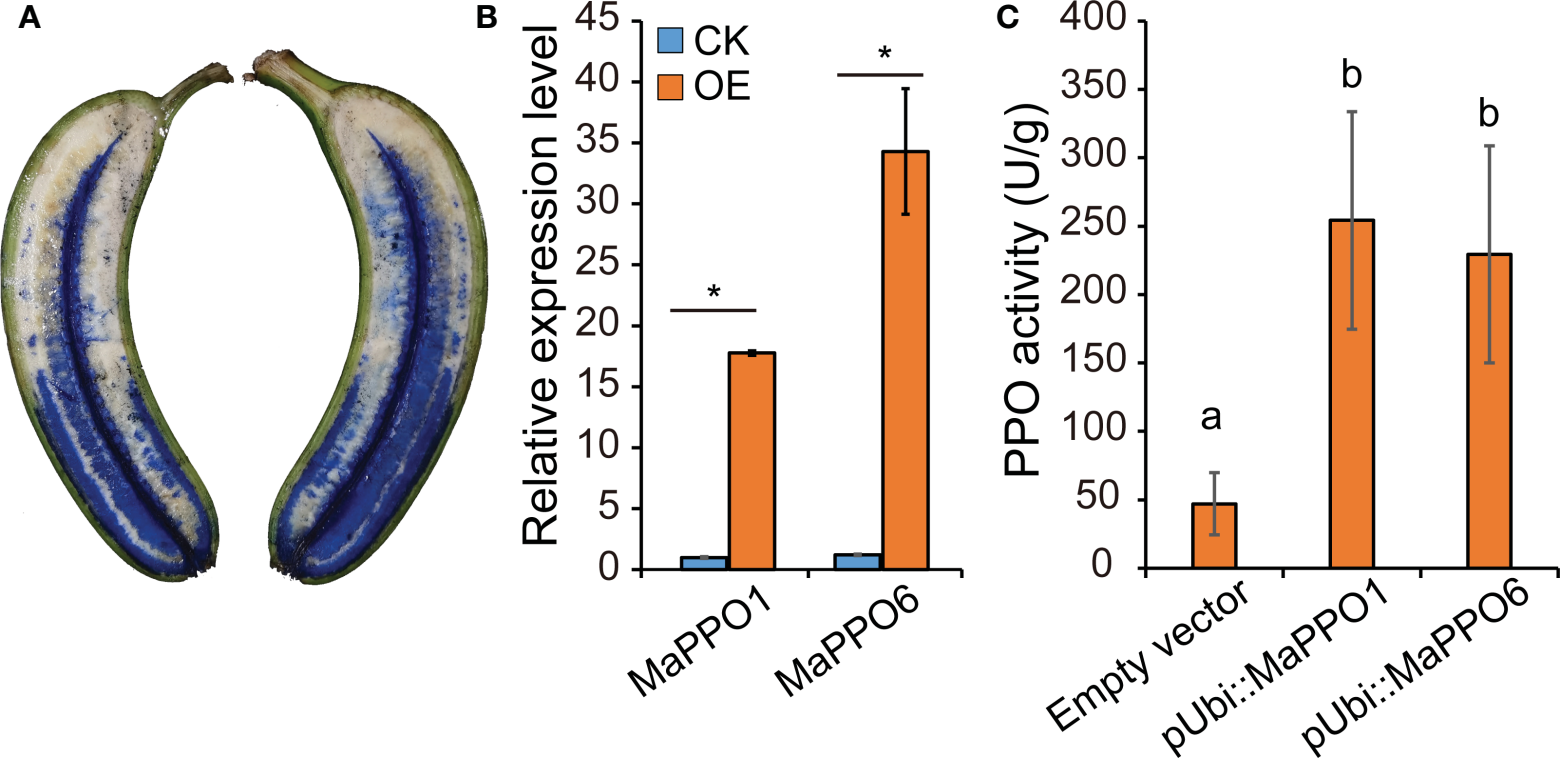
Transient expression analysis of *MaPPO* genes in banana fruit **(A)** Presentation of the delivery of *Agrobacterium* solution into banana pulp through the distal end of the fruit. **(B)** qRT-PCR analysis of the expression level of *MaPPO* genes. P-values were determined using Student’s t-tests. *P < 0.05. **(C)** Polyphenol oxidase activity of *MaPPO*-overexpressing fruit. Different letters indicate significant differences (P < 0.05) examined by one-way ANOVA.

To determine whether *MaPPO* genes encoded an active polyphenol oxidase enzyme, *MaPPO1/6/7/10* were chosen for prokaryotic expression in *Escherichia coli*. Recombinant MaPPO proteins with a His-tag were isolated and purified. The molecular weight of these proteins was verified with Western blot analysis using an anti-His antibody (**Figure 8**), and the molecular mass of recombinant PPOs was consistent with the predicted molecular mass, indicating successful expression in the prokaryotic expression system. The enzyme activity assay indicated that MaPPO1 had the highest activity among the examined MaPPO proteins, followed by MaPPO6, MaPPO7, and MaPPO10, and MaPPO1 yielded a PPO activity 7-fold higher than that of MaPPO10. The results indicate that MaPPO1 and MaPPO6 might contribute to the main PPO activity in banana fruit. To further examine the *in vivo* activity of MaPPO proteins, MaPPO1 and MaPPO6 were transiently overexpressed in banana fruit (**Figure 9**). In the MaPPO-overexpressing fruit, the expression level of *MaPPO1* and *MaPPO6* was about 20 times and 35 times that in the control fruit, suggesting successful expression of target genes (**Figure 9B**). PPO activity analysis indicated that MaPPO1- and MaPPO6-overexpressing fruit had 6- and 5-fold higher PPO activity than the control fruit, implying that they are active polyphenol oxidase enzymes in banana (**Figure 9**).

## Discussion

As widespread copper-containing metalloenzymes in plants, PPOs play important roles in plant growth and development, stress resistance, and stress tolerance, and they widely exist in land plants, fungi, and some bacteria. The member numbers of the *PPO* gene family varied with species and were not directly involved in the size of the genome. No *PPO* genes were identified in *Arabidopsis*, *Brassica napus*, or some green algae, implying that PPOs are likely not necessary for primary metabolic function; rather, they are involved in secondary metabolism and ecological adaptation (Tran et al., 2012).

Although several *MaPPO* genes of banana have been reported to be available in the banana genome (Gooding et al., 2001), detailed information on these *MaPPO* genes is missing, and the role of *MaPPOs* is still not clear. In this research, we identified 10 *PPO* genes in banana with the available whole genome sequence. Most *MaPPOs* had one intron, while *MaPPO1* had two introns, and *MaPPO2/5* had no introns (**Figure 2B**). Introns have been reported to regulate transcription, and intron-lacking genes might exhibit the rapid expression of mRNA in response to various stressors (He et al., 2021). This implies that *MaPPO2/5* genes are transcribed faster under certain conditions to form mRNA.

Except for MaPPO4, all MaPPO proteins contained three conserved domains (KFDV, tyrosinase, and DWL). They may offer similar and conserved biological functions (**Figure 2D**). Among them, *MaPPO6/7/8/9* genes were located on chromosome 8 (**Table 1**), had high sequence similarity, and clustered together in the same group (**Figure 1A**). We hypothesized that *MaPPO7/8/9* originated from *MaPPO6* by gene duplication. This is supported by the expression data (**Figure 4**). Among them, *MaPPO6* had the highest expression, *MaPPO7* exhibited trace expression, and the transcripts of *MaPPO8/9* were not detectable.

Generally, plant PPOs are primarily localized in plastids or chloroplasts (Tran et al., 2012), which are determined by the N-terminal targeting signal, a chloroplast transit peptide (cTP) responsible for the translocation of PPO proteins to the thylakoid lumen (Koussevitzky et al., 2008). In contrast, aureusidin synthase 1 (AmAS1) in *Antirrhinum majus* and PtrPPO13 in *Populus trichocarpa* have been reported to be localized in the vacuole (Ono et al., 2006). Nevertheless, in potato tubers and tea, PPOs were found in almost all subcells and organelles (Vámos-Vigyázó and Haard, 1981; Zhang and Sun, 2021), and a chalcone synthase in wine grapes was also localized in multiple organelles, predominantly in the plastid, rough endoplasmic reticulum (ER), cytoplasm, vacuole, and cell wall (Tian et al., 2008). In banana, MaPPO1 and MaPPO7 were distributed in chloroplasts, and MaPPO6 was localized both in chloroplasts and in ER. MaPPO10 is an ER-localized PPO, implying that it might be related to the biosynthesis of secondary metabolites because chalcone synthase, the first enzyme of the flavonoid pathway, was found to be localized in the cytosol and ER (Kaintz et al., 2014). These data suggest that the location of PPO varies with plant species and that PPOs might function differently with various subcellular locations.

*PPO* genes of most plant species are present as a multigene family, except for several *PPO*-lacking plant species (Tran et al., 2012). The qRT-PCR results and transcriptome data indicated that the transcript levels of *MaPPOs* are distinctly regulated in various tissues, fruit development, or during fruit ripening, and 4 of the 10 *MaPPO* genes were not detectable in the examined tissues using qRT-PCR. This is consistent with that of other plant species (He et al., 2021). The expression pattern of *MaPPO6* and *MaPPO7* was similar to that of *BPO1* in a previous report (Gooding et al., 2001) and was not detected in mature leaves but was present in young leaves and stems. Moreover, the expression of detected *MaPPOs* genes, except *MaPPO1*, was consistent with that in other plant species and generally decreased during fruit development and maturation (**Figure 5B**, fruit development), suggesting that fruit browning during ripening might result in a pre-existing PPO that is expressed in early fruit development. Our data revealed that *MaPPO1* is specifically expressed and is the highest-expressed *PPO* gene in the fruit tissue of all examined *PPO* genes. The proteome data also indicated that MaPPO1 is the most abundant PPO protein during fruit ripening, followed by MaPPO6 (**Figure 4B**). Therefore, MaPPO1 and MaPPO6 might be the main contributors to the PPO activity that causes fruit browning (**Figures 4A, B**, control; **Figure 5A** 15 d; **Figure 6A**, 0 d). Moreover, their high PPO activity *in vivo* and *in vitro* and their chloroplast location also supported this (**Figures 7**–**9**). We speculated that the functions of the two *MaPPO* genes may be redundant or partially overlapping. Therefore, knockout of *MaPPO1* or *MaPPO6*, or both, in banana could result in reduced detrimental oxidative browning in fruit tissues. In subsequent research, we will develop a *PPO*-mutated banana using our group’s established genome editing approach, and we believe that the analysis of mutant plants will improve our understanding of the role of *MaPPOs* in banana.

## Conclusions

In summary, 10 *MaPPO* genes were identified through genome-wide analysis based on released genome data. Analysis of gene structures, motifs, and sequence features revealed the conservation and divergence of *MaPPOs*. Expression analysis indicated that *MaPPO1* and *MaPPO6* exhibit high transcript abundance in mature green fruit, paralleled by the protein content data, and *MaPPO1* was preferentially expressed in fruit tissues. All detectable *MaPPO* genes in mature green fruit showed reduced expression trends during fruit ripening, except *MaPPO1*. Moreover, MaPPO1 and MaPPO6 showed chloroplast localization in tobacco leaf cells, indicating that MaPPO1 and MaPPO6 play important roles in fruit browning. In addition, the PPO activity assay of MaPPO *in vivo* and *in vitro* supported the idea that MaPPO1 and MaPPO6 might offer major PPO activity in fruit tissues. This research reveals that MaPPO1 and MaPPO6 are major contributors to fruit browning during fruit ripening, which will further our understanding of the function of *MaPPO* genes and provide candidate genes for developing new varieties with low fruit browning in the future.

## Data availability statement

The datasets presented in this study can be found in online repositories. The names of the repository/repositories and accession number(s) can be found below: https://www.ncbi.nlm.nih.gov/genbank/, PRJNA598018.

## Author contributions

FB and GY conceived and performed the original research project. FQ, CH, and WH performed the experiments. OS, QY, TXD, GD, HG, CL, and TD designed the experiments and analyzed the data. FQ and FB wrote the manuscript with contributions from all authors. FB and GY supervised the experiments and revised the writing. All authors contributed to the article and approved the submitted version.

## Supplementary material

Supplementary file 1 Sequence information of *MaPPO* genes.
