# Supplementary material for: Genome-wide analysis of the polyphenol oxidase gene family reveals that MaPPO1 and MaPPO6 are the main contributors to fruit browning in Musa acuminate
Source: Front Plant Sci. 2023 Feb 14;14:1125375. doi: 10.3389/fpls.2023.1125375 (PMC9971926; doi:10.3389/fpls.2023.1125375)
Supplement: Supplementary file 1 — Sequence information of MaPPO genes. [file Table_1.docx]

**>MaPPO1-CDS**

ATGGCTTCTATCTCGCAGCTAATCACTACAAGCATCCCCACCACCTTTCCTCTCTCCTCTTCATGCCCCTTCTCCCCTCCGAGAACCACGGTATCGATCTCCGGCTTCAAACACCACCACCACGTTTCCCCCATCTCATGCTCCTCCAGAGACCACAACCAACCCCTCGTTGATCCCACAGTCGATCGCCGCCACGTCCTCGTCGGACTAGGCAGCCTCTACGGCGCCTCCGCTGCTCTAACGTCCCTCCACGAGGCCAGCGCGGCACCGATCGCGGCGCCCGACCTGTCCGCATGCGGGCCGGCTGACCTCCCTCCGGATGCCACTCCGACGAACTGCTGCCTGCCGTCCGCGGGCGACGCGACCGAGTTCGTCATTCCCGACCCGTCCTCGCCCTTGAGGGTGCGCCCGGCGGCCCACTCGGTCGACAACGATTACATAGCTAAGTTCGCGAAGGGCGTCGCTCTCATGAAGGCGCTTCCGGCCGACGACCCCCGGAACTTCACTCAGCATGCCAACGTGCACTGCGCCTACTGCGACGGGGCGTACAGCCAAGTCGGCTTCCCGGACCTTGAGCTCCAGGTGCACAACTCATGGCTCTTCCTGCCATGGCACCGCTGCTACCTCTACTTCTTCGAGAGAATACTCGGGAAGTTGATCGGCGACGACAGCTTCGCGATTCCGTTCTGGAACTGGGACGCCCCTGACGGGATGCGATTGCCAGCGATGTACGTGGATCCCACGTCGCCGCTTTACGATCCCCTAAGGGATGCACAGCATCAGCCGCCGACGTTGGTGGATTTGGACTTCGGAGGGATCGATCCTTCTTTCAGTGATAAGCAGCAGATTGATCACAACCTCAAGGTTATGTACAGGCAGATCGTCTCGAATGCACCGACACCGAGGCTCTTCTTCGGAAACCCGTACCGAGCCGGCGACAATCCGAACCCCGGTGGCGGCTCGCTTGAGAACGTCCCCCACGGACCGGTCCACGTCTGGACCGGCGACCGCAGCCAGTCGGAACTGGAGGACATGGGCAACCTGTACTCCGCCGCTCGCGACCCCGTCTTCTTCGCCCACCACTCCAACATCGACCGCATCTGGAACGTGTGGAAGGGTCTCGGTAGCCGGCGCAAGGACCTGGCCGACCCCGACTGGCTCGACGCCTCCTTCGTCTTCTACGACGAGAACGCCAACCTCGTCAAGATCCGAGTTCGCGACTGCATCGACTCAGACAAGCTACGCTACGAGTACCAGGACGTCGGTAACCTATGGCTCAACACACGCCCGACGGTGACGTCCGGAGTGAGGCCGAGAGTGGCCGGAGTGGCGCATGCAAACGTGGTGGAGCCGAAGTTTCCGATAAAGTTGGACTCGGTGGTGACTGCCAAGGTGAAGAGGCCAAAGGCGGCGAGGACCAAGGAGGAGAAGGAGGAGAAGGAGGAGGTGCTGGTGGTTGAAGGGATCGAGCTGGATCGAGACGTGCACGTCAAATTCGACGTGTTCGTGAACGTGACCGATCACGGGAAGGTCGGGCCGGGGGGCCGGGAGCTCGCCGGGAGCTTCGTGAACGTGCCTCACAGGCACAAGCATGACAAGATGAGCAAGCAGCTGAAGACCAGGCTGCAGCTGGGCTTGACTGAGCTGTTGGAGGATCTCAAGGTTGAAGGAGATGGGAGCATCATGGTGACTTTGGTGCCGAGGCAGGGGAAGGGGAAGGTGAAGGTTAGCAGTCTCAAGATCGAGTTAGTTGATTGA*

**>MaPPO2-CDS**

ATGGCCGGCCTTCCTTATTCGGCTCCTCACCCTGCCACCATCTCCGCTTCCTCCAACTCCTTTGCATGCCCCTTCCGCAGCAAGGGGCTTGTCTTCCCCTACCCTACCAGAAGAGCACTCCATGTTCGTCCCAACATCGCATGCAAGGCAGGCGAGGAGCACGAGATCGCCGCTAAGGTCGACCGACGCGACGTGCTCGTGGGCCTCGGTGGGCTCTGCGGAGCCGCCGCTGGCCTTGGCGGGTTCGATAAAGCCGCCCTCGCTAACCCCATTCAGGCCCCTGATCTCTCCAAGTGCGGCCCTGCCGACCTCCCCACCGGCGTGCCAATCGTCAACTGCTGCCCGCCCTACCGTCCCGGCAAGAAGATTGTGGATTTCAAGCGGCCGTCGCCGTCCTCCCCCCTCCGCGTCCGCCCCGCCGCCCAGTTGGTTGACCCCGAGTACCTGGCCAAGTACAAGAAGGCCATCGAGCTCATGAAGGCGCTCCCGGCCGACGACCCTCGCAACTTCATGCAGCAGGCCGACGTCCACTGCGCCTACTGCGACGGCGCTTACGACCAGATCGGCTTCCCCGACCTTGAGATCCAAGTCCACAACAGCTGGCTCTTCTTCCCCTGGCACCGCTTGTACCTCTACTTCAACGAGAGGATCCTCGGCAAGCTCATCGGCGACGACACCTTCGCGCTCCCTTTCTGGAACTGGGACGCACCCGGCGGAATGATGCTGCCGTCGATCTACGCCGACCCTTCGTCGCCCCTCTACGACAAACTTCGCGACGCCAAGCACCAACCACCTGTCCTTGTCGACCTCGACTACAATGGAACCGACCCAACCTTCCCCGACGACCAGCAAATCGATCACAACCTCAAGATCATGTACCGCCAAGTCTTCTCCAACGGCAAGACGCCGTTGCTGTTCTTAGGCTCAGCTTACCGTGCCGGTGACCAGCCTAACCCCGGCGCGGGATCCATCGAGAACATGCCGCACAACAACGTGCACTTGTGGACCGGCGACCGCACCCAGCCCAACTTCGAGAACATGGGCACCTTCTACGCGGCGGCGCGCGACCCCATCTTCTTCGCCCACCACGCCAACATCGACCGAATGTGGTACCTGTGGAAGAAGCTCAGCAGGAAGCACCAGGACTTCAATGACTCGGACTGGCTCAAAGCTTCCTTCCTCTTCTACGACGAGAACGCCGACTTAGTTCGGGTCACGGTCAAGGACTGCTTGGAGACCGAGTGGCTGCGCTACACGTACCAAGACGTGAAGATCCCATGGGCGAACACCCGACCGACTCCCAAGCTCGCCAAGGCGAGGAAAGCCGGCAGCAGATCGCTGAAAGCCACCGCGGAGGTGCAGTTCCCTGTGACGCTGGAATCCCCGGTCAAAGTGACGGTGAAGAGGCCCAAGGTGGGGAGGAGCGGCAAGGAGAAGGAAGATGAGGAGGAGATACTCATAGTGGAGGGGATCGAGTTCGACCGCGACTACTTCATCAAGTTCGACGTCTTCGTGAACGCGACGGAGGGCGACGGCATCACGGCCGGGGCCAGTGAGTTCGCCGGCAGCTTCGTGAACGTCCCGCACAAGCACAAGCACCGCAAGGATGAGAATAAGCTGAAGACGAGGCTGTGTCTGGGAATCACCGACCTGCTCGAGGACATCGGCGCGGAGGACGACGACAGCGTGCTCGTCACCATCGTGCCGAAGGCAGGCAAAGGAAAGGTGTCCGTCGGCGGTCTTCGGATTGACTTTTCCAAGTGA*

**>MaPPO3-CDS**

ATGGCAGAGATCGGCAATCCAAATGAAAACAACATCTCAATATTTGCCGACCGCAAGCCGCCGAAACGTCTCGTGTTGCCATTCGGCGTCCGACCACCAGTGGCAGAGGCACTGACAAAGGTTGATGCAAGTTTCTGCGACCCCAAGAACAACAACGAGTGTATAAACTTAGGATCCCAAGGAGGCGAGTGCGCCGCCAACTGCTGCCTGCCCATCCGTCCCGGTGCGAAGATTGTGGATTTCAAGCGGCCATCGCGGTCATCCCCCCTCCGCGTCCGCCCCGCCGCCCACTTGGTCGACCCCGAGTACCTGGCCAAGTACAAGAAGGCCATCGAGCTCATGAAGGCGCTCCCGGCAGACGACCCTCGCAACTTCATCCAGCAGTCCAACGTCCACTGCGTTCACTGCGACAGCATCCCCGACCATGACATCCAAGTCCACCAGAACTGGTTCTTCTACCCCTGGCACCGCTGGTACCTCTACTTCAACGAGAGGATCCTCGGCAAGCTCATCGGCGACGACAACTTCACGCTCCCTTTCTGGAACTGGGACTCGCTCGGCGGAATGATGCTGCCGTCGATCTACGCCGACCCTTCGTCGCCCCTCTACGACAACCTTCGCGACGCCAAGCACCAACCTCCTTTCCTTGTCGACTTCGACTTCAATGAAACCGACCCAGGCTTCACCGACGCCCAGCAAATCGATCACAACCTCAAGATCATGTACCGCCAATTCTTCTCCAACGGCAAGAAGCCGTTGCTGTTCTTAGGCTCAGCTTACCGCGGGGGCGACAAGCCTAACCCCGGCGGGGGCTCCGTCGAGAACACGCCGCACAACAACGTGCACACGTGGACCGGCGACCGTACCCGTCCCGACTTCGAGGACATGGGCACCTTCTACTCGGCGGGACGCGACCCCATCTTCTTCGCCCACCACGCCAACATCGATCGCATGTGGTCCCTGTGGAAGAAGCTCAGCCGTAAGCACCGGGACTTCAATGACTCGGACTGGCTGAAAACTTCCTTCCTCTTCTACGACGAGAACGCCGACTTAGTTCGGGTCAAGGTCAAGGACTGCTTGAAGACCCGGTGGCTGCGCTACAAGTACCAAGACGTGGAGATCCCATGGGTGAAAGCCCGACCGACTCCCAAGCTCACCAAGGCGAGGAAAGCCGCCAGCGGATCGCTGAAACCCACCGCGGAGGCGCAGTTCCCTGTGACGCTGGAATCCCCGGTCAGCGCGACGCTGAAGAGGCCCAAGGTGGGGAGGAGCCGCAAGGAGAAGGAAGAGGAGGAGGAAGTACTCATAGTGGAGGGGATCGAGTTCGACCGCGACCAGTTCATCAAGTTTGACGTCATCGTGAACGCGACGGAGGGCGACGGCATCACGCCCGCGGACAGCGAGTTCGCCGGCAGCTTCGTGAACGCCCCGCACAGGCACAGGCACCTCAAGGAGGAGAACAAGGGGACGACGAGGCTGTGTCTGGGGATCACCGACCTGCTCGAGGACATCGGCGGGGAGGCCGACGACGGCGTGCTCGTCACCATCGTGCCCAAGGCAGGCAAGGGCAAGGTTTCCGTCGGCGGTCTTCGGATTGACTTCACCAAGTGA*

**>MaPPO4-CDS**

ATGTCCACCGGAGCGGCCATCGTGCTGAGCGCCACTGCCGCCGGCGCTGCCTCTGCGTGCCTCCTCCAGCGACAGCGCTCCCGCCGCCTCCCTCGCGTGTCATGCCACCGGGGAAGCCACGATCGAAGCGAGGATCCTCGGCCGACCCTGCTGCAACGGCGAGGCTTGCTGATCGTCGGTTTGGGAGGGCTATGCAGCGTGGCCGCGGGGCCGATCGTGCTCGCGGAGCCGGTGGATTCGTCCAAGCTAACGGCGATGGAGACCGAGGACGAGCTGCTGCGCGTGGAGGAGTTCGGAACGCAGCCCCGGAGGTTGGACCCCACCAGGCCGCTCCGGGTGTTGGTGGCGAGGCCCAAGAAGAGCCGCACCAAGGCGGAGAAGGAGCAGGAGGTGGAGGTACTTCAGATCAACGGCATACGGGTGGATCCGAGAAGCGCAACTCGCTTCGATGTCTACATCGCTGCCCCGCGCGGCGACCTCGCAGGGCCGGGTCTTGGGGAGTTCGCAGGCATCTTCCTGAAGCTGCCGTACAAAAGGGAGGACAGCGTCGTCGTGAGGACGGCAGGTCTCAAGCTGGGCCTCACCTCACTGTTGGACGACATGAATGCCGATGACGCTGAAAAGCTGGTGGTCTCTTTGGTTCTTCGTGCGGGCGACGTTACTGTGGGAGATATTAGTATCGATCTGAGGAAGACTGATATGGCCAGAGATATGTAG*

**>MaPPO5-CDS**

ATGGTCAGCCTTCCTAAAGCTACTCTTCCTCTCTCCTCCCTCTCCCCTCCCTCCAACTCCAACTCCAACTCCAACTCCAACTCCTTTGCATGCGCCTTCCATTTTTCTTACCCTGATAGAAGACGCCATGCCCACTCCAAGATCTCATGCAAAGCTAGCGATGAGCATGAGATGACTGCAAATGCCAAGCTCGACCGCCGCGATGTGCTCGTTGGCCTCGGCGGGCTTTGTGGAGCCGCTGCCGGCCTCGGGATCGACGGTAAGGCCCTCGGAAATCCCATCCAGGCGCCTGATCTTACCAAGTGCGGCCCCGCCGATCTACCCAAAGGTGCAACGCCCACCAACTGCTGCCCGCCTTATTTTCCCGACAAGAAGATTATCGATTTCAAGCGTCCGCCGAATTCGTCACCCCTCCGTGTCCGCCCGGCCGCCCACTTGGTCGACTCCGACTACCTGGACAAGTATAAGAAGGCGGTGGAGCTCATGAGAGCACTGCCGGCCGACGATCCGCGCAACTTCATGCAGCAGGCCAATGTCCACTGCGCTTACTGTGATGGCGCCTACGACCAGATCGGCTTCCCCAACCTCGAGCTCCAAGTCCACAACTCCTGGCTCTTCTTCCCTTGGCACCGCTTCTACCTCTACTTCCACGAGAGGATCCTCGGCAAGCTCATAGGCGACGACACTTTCGCCCTTCCTTTCTGGAACTGGGACGCGCCCGGCGGCATGAAGCTGCCGTCGATCTACGCCGATCCTTCGTCCTCGCTCTATGACAAGTTTCGCGACGCCAAGCACCAGCCGCCGGTCCTCGTCGACCTCGACTACAACGGAACCGACCCTAGTTTCACCGACGCAGAGCAGATCGATCAGAACCTCAAGATCATGTACCGGCAGGTGATCTCCAACGGCAAGACGCCGTTGCTGTTCTTAGGCTCGGCTTACCGTGCCGGCGACAACCCAAACCCCGGCGCGGGCTCGCTCGAGAACATACCACACGGCCCCGTCCACGGGTGGACTGGCGACAGAAACCAACCCAATCTCGAGGACATGGGCAACTTCTACTCCGCGGGGCGCGACCCTATCTTCTTCGCCCACCATTCAAACGTCGACCGCATGTGGTACTTGTGGAAGAAGCTCGGCGGGAAGCATCAGGACTTTAACGATAAGGACTGGCTCAACACCACCTTCCTCTTCTACGACGAGAATGCTGACTTAGTTCGAGTCACCCTCAAGGACTGCTTGCAGCCGGAGTGGCTTCGTTACGATTACCAAGACGTCGAGATCCCGTGGCTGAAGACCCGGCCGACTCCCAAAGCCTTGAAGGCGCAGAAAACCGCAGCGAAAACACTGAAAGCTACAGCAGAGACGCCGTTCCCGGTGACGCTGCAATCCGCGGTGAGCACGACGGTGAGGAGGCCCAAGGTATCGAGGAGCGGCAAGGAGAAGGAAGAGGAAGAGGAGGTCCTCATCGTGGAGGGGATCGAGTTCGACCGCGACTACTTCATAAAGTTCGACGTCTTCGTGAACGCCACCGAGGGTGAGGGCATCACGCCGGGCGCCAGCGAGTTCGCGGGCAGCTTCGTCAACGTCCCGCACAAGCACAAGCACAGCAAGAAGGAGAAGAAGCTGAAGACGAGGCTCTGCCTGGGGATCACTGACCTGCTCGAGGACATCGGGGCGGAGGACGACGACAGCGTGCTCGTCACCATCGTCCCGAAAGCCGGAAAGGGCAAGGTGTCGGTCGCCGGCCTCCGCATCGATTTCCCAAATTGA*

**>MaPPO6-CDS**

ATGTCCCTGCTGTTGAACTCTAGCTTCACCGGTGCTTCCTCTGCATGCCTTCTCCAACGGGAAAGGTCCCGCCGCCGCCGCCTCCACGTCCCTGGCGTGACTTGTCGCCAGGGCAGTAATGGTGACCGCAGCGATGCCGCCCGCCAGCAGCAGTCGCCGCCGCTGCTGGATCGGCGCGACATGCTGTTGGGTTTAGGAGGGCTTTACGGCGTGACCGCAGGACCCAAGGTTCTGGCGGCGCCGATAATGCCGCCGGATCTGTCCAAGTGCTACCCTGCCACCGCACCTGCCCTCGACAACAAATGCTGCCCGCCTTACGACCCCGGCGAGACGATCTCGGAGTACAGCTTCCCTGCTACGCCCCTCCGGGTGCGGCGGCCGGCCCATATCGTGAAGGACGATCAGGAGTATATGGACAAGTACAAGGAGGCAGTGAGGAGGATGAAGAATCTGCCGGCAGACCACCCTTGGAACTACTACCAGCAGGCGAACATCCACTGCCAGTATTGCAACTACGCCTACCACCAGCAAAATACCGACGACGTGCCCATCCAGGTCCACTTCAGCTGGATCTTCCTCCCATGGCACCGCTACTACCTCCACTTCTACGAAAGGATCCTCGGCAAGCTCATCGACGACGACACCTTCACCATCCCATTCTGGAACTGGGACACCAAGGACGGCATGACGTTCCCCGCCATCTTCCAGGATGCGGCATCCCCGCTGTACGACCCGAGACGCGACCAACGCCACGTCAAGGACGGCAAGATCCTCGACCTCAAGTACGCCTACACCGAAAACACTGCATCCGACAGCGAGATCATACGGGAGAACCTCTGCTTCATACAGAAGACGTTCAAGCACAGCCTGTCGCTGGCGGAACTGTTCATGGGGGATCCCGTGCGCGCGGGGGAGAAGGAGATCCAGGAGGCTAATGGGCAGATGGAAGTCATCCACAATGCGGCGCACATGTGGGTCGGAGAGCCGGACGGATACAAGGAAAACATGGGGGACTTCTCCACCGCCGCCCGCGATTCTGTTTTCTTCTGCCACCATTGCAATGTCGACCGCATGTGGGACATCTACCGCAACCTCCGCGGCAACCGCGTCGAGTTCGAAGACAAAGACTGGTTGGACAGCACCTTCCTCTTCCACGACGAGAACGAACAGCTCGTCAAAGTCAAGATGAGCGACTGCCTCAACCCGACCAAGCTTCGGTACACGTTCGAGCAAGTTCCCCTCCCATGGCTGGGCAAAATCAATTGCCAGAAGACGGCAGAGACGAAGTCCAAGGCCACGACGGAGCTGTCGCTGACGCGCGTGAACGAATTCGGGACGACGGCCCAGGCACTCGACGCGAGCAACCCGCTGCGGGTGATCGTGGCAAGGCCGAAGAAGAACCGCAAGAAGAAGGAGAAGCAAGAGAAGGTGGAGGTGATTCAGATCAAGGATATTCAGGTGACCACCAACGAGACAGCTCGCTTCGACGTCTACGTCGCGGTTCCTTACGGTGACCTCGCCGGACCCGACTACGGCGAGTTCGCTGGCAGCTACGTGAGGCTGGCGCATAGGATGAAGGGAAGCGAGGGGACCGAAGTGCAGGGCCCCAAGAAGAAGGGAAAGCTCAAGCTAGGTATTACGCCACTGCTCGAGGACATCGATGCTGAGGACGCCGACAAGTTGGTGGTCACCCTGGTTCTCCGCACCGGGAGCGTCACCGTGGGCGGAGTTTCCATCAATCTCCTGCAGACAGATTCTACCGCCGCCATCTAA*

**>MaPPO7-CDS**

ATGTCCCTGCTGTTGAACTCTAGCTTCACCGGTGCTTCCTCTGCATGCCTCCTCCAACGGGAAAGGTCCCGCCGCCGCCGCCTCCACGTCCCTGGCGTGACATGCCGCCAGGGCAGTAATGGTGACCGCAGCGATGCCGCCCGCCAGCAGCAGTCGCCGCTGCTGCTGGATCGGCGCGACATGCTGTTGGGTTTAGGAGGGCTTTACGGCGTGACCGCAGGACCCAAGGTTCTGGCGGCGCCGATAATGCCGCCGGATCTGTCCAAGTGCTACCCTGCCACTGCACCTGCCCTCGACAACAAATGCTGCCCGCCTTACGTCCCCGGCGAGACGATCTCGGAGTACAGCTTCCCTGCTACGCCCCTCCGGGTGCGGCGGCCGGCCCATATCGTGAAGGACGATCAGGAGTATATGGACAAGTACAAGGAGGCAGTGAGGAGGATGAAGAATCTGCCGGCAGACCACCCTTGGAACTACTACCAGCAGGCGAACATCCACTGCCAGTATTGCAACTACGCCTACCACCAGCAAAATGCCGACGACGTGCCCATCCAGGTCCACTTCAGCTGGATCTTCCTCCCATGGCACCGCTACTACCTCCACTTCTACGAAAGGATCCTCGGCAAGCTCATCGACGACGACACCTTCACCATCCCCTTCTGGAACTGGGACACCAAGGACGGCATGACGTTCCCCGCCATCTTCCAGGATGCGGCATCCCCGCTGTACGACCCGAGACGCGACCAACGCCACGTCAAGGACGGCAAGATCCTCGACCTCAAGTACGCCATCACCGAAAATGAAAGCACTGCATCCGACAGCGAGATCATACGGGAGAACCTCTGCTTCATACAGAAGACGTTCAAGCACAGCCTGTCGCTGGCGGAGCTGTTCATGGGGGATCCCGTGCGCGCGGGGGAGAAGGAGATCCAGGAGGCTAACGGGCAGCTGGAAGTCATCCACAATGCGGTGCACAGTTGGGTCGGAGAGCCGAGCGGAAACTATGAAGACATGGGCTACTTCTCCACCGCCGCCCGCGATTCTGTTTTCTTCTGCCACCATTGCAATGTCGACCGCATGTGGGACATCTACCGCAACCTCCGCGGCAACCGCGTCGAGTTCGAAGACAACGACTGGTTGGACAGCACCTTCCTCTTCCACGACGAGAACGAGCAGCTCGTCAAAGTCAAGGCAAGTCAGATGCTGGACTGCCTCAACCCGACCAAGCTTCGGTACACGTTCGAGCAAGTTCCCCTCCCATGGCTGGGCAAAATCAATTGCCAGAAGACGGCAGAGACGAAGTCCAAGGCCACGACGGAGCTGTCGCTGAATCGCGTGAACGAATTCGGGACGACGGCCCAGGCACTCGACGCGAGCAACCCGCTGCGGGTGATCGTGGCAAGGCCGAAGAAGAACCGCAAGAAGAAGGAGAAGCAAGAGAAGGTGGAGGTGATTCAGATCAAGGATATTAAGGTGACCACCAACGAGACAGCTCGCTTCGACGTCTACGTCGCGGTCCCTTACGGTGACCTCGCCGGACCCGACTACGGCGAGTTCGTGGGCAGCTACGTGAGGCTGGCGCATAGGATGAAGGGAAGCGAGGGGACCGAAGTGCAGGGCCCCAAGAAGAAGGGAAAGCTCAAGCTAGGTATTACGCCACTGCTCGAGGACATCGATGCTGAGGACGCCGACAAGTTGGTGGTCACCCTGGTTCTCCGCACCGGGAGCGTCACCGTGGGCGGAGTTTCCATCAATCTCCTGCAGACAGATTCTACCGCCGCCATCTAA*

**>MaPPO8-CDS**

ATGTCTCTCCTGTTGAACTCTAGCCTCACCGGAGCTTCCTCTGCATGCCTCCTCCGTCGAGAAAAGTGCCGCCGCCGCGGCCGCGGTCACGTCCACGGCGTGACATGCCGCCAGGGGGGTAATGATGACCGCAGAGACGCCGCCCGGCAGCAGCGGTCCCGGTTGCTGCTGGATCGGCGCGACATGCTGTTGGGGGGGTTAGGAGGGCTTTACGGCGTGACCGCAGGGCCCAAAGTTCTGGCGGAGCCGATAATGCCGCCTGATCTGTCGAAGTGCCACGATGCCAACGCACCTGCCCTCCACAACCACTGCTGCCCGCCTTACAGCGGCAGCGAGACGATCTTGGAGTACGACTTCCCCGCTGCGCCCCTCCGGGTGCGGCAACCGGCCCACCTCGTGAAGGATGATCAGGAGTATATGGACAAGTACAAGGAGGCCGTGAGGAGGATGAAGAACCTGCCGGCAGAACACCCTTGGAACTACTACCAGCAGGCGAACATCCACTGCCAGTATTGCAACGACGCCTACTACCAGCAAAATACCGACGACGTGCCCGTCCAGGTCCACTTCAGCTGGATCTTCCTCCCCTGGCACCGCTACTACCTCCACTTCTACGAGCGGATCCTCGGCAAGCTCATCGACGACGACACCTTCACCATCCCCTTCTGGAACTGGGACACCAAGGACGGCATGACGTTCCCCGCCATCTTCCAGGATGCGGCATCCCCGCTGTACGACCCGAAACGCGACCAACGTCACGTCAAGGACGGCGCGATCCTCGACCTCAAGTACGCCTACACCGAAAACACTGCATCCGACAGCGAGATCATACGGGAGAACCTCTGCTTCATACAAAAGACGTTCAAGCACAGCCTGTCGCTGGCGGAGCTGTTCATGGGGGATCCCGTGCGCGCGGGGGAGAAGGAGATCCAGGAGGCAAACGGGCAGCTGGAAGTCATCCACAATGCGGCGCACATGTGGGTCGGAGAGCCGGACGGATACAAGGAAAACATGGGCGACTTCTCCACCGCCGCCCGCGATTCTGTTTTCTTCTGCCACCATTGCAATGTCGACCGCATGTGGAACATCTACCGCAACCTCCGCGGCAACAGCGTCGAGTTCAAAGACAAAGACTGGTTGGACAGCACCTTCCTCTTCCATGACGAGAACGAGCAGCTCGTCAAAGTCAAGATCCAGGACTGCCTTAACCCGACCAAGCTTCGGTACACGTTCGAGCAAGTTCCCCTCCCATGGCTGGGCAATATAAATTGCCAGAAGACGGCAGAGACGAAGTCCAAGTCCACGGCAGAGCTGTCGCTGAAGCGGGTGGGCGAATTCGGGACGACACCCAAGGCGCTCGACGCGAGCAACCCGCTGCGGGTGATCGTGGCAAGGCCGAAGAAGAACCGCAATAAGATGGAGAAGCAAGAGAAGGTGGAGGTGCTCCAGATCAAGGATATTAAGGTGACCACCAACGAGACAGCTCGCTTCGACGTCTACGTCGCCGTTCCTTACGGTGACCTCGCCGGACCCGACTACGGCGAGTTCGTGGGCAGCTTCGTTAGGCTGGCGCATAGGAAGAAGGGAAGCGACGGGAACGAAGAGCAGGGCCCCAAGAAGAAGGGAAAGCTCAAGCTGGGTATTACGGCGCTGCTCGAGGACATCGATGCTGAGGACGCCGACAAGTTGGTGGTCACCCTGGTTCTCCGCACCGGGAGCGTCACTGTGGGTGGAGTTTCCATCAAACTCCTGCAGACAGATACTCCCGCCGTCATCTAA*

**>MaPPO9-CDS**

ATGGCTCTCCAGTTGAACTCTAGCTTCACCGGAGCTTCCTCTGCATGCCTCCTCCATCGGGAAAGGTCCCGCCGCCTCAACGTCCCTGTCGTGACATGCCGCCAGGGGAATAATGATGATCGCAGCGATGCCGCTCGCCAGCAGAAATCCCCGTCACTACTGGATCGGCGCGACATGCTGCTGGGGTTAGGAGGGCTTTATGGCTTGACCGCAGGACCCAAAGTTCTGGCGAAGCCGATAATGCCGCCTGATCTGTCCAAGTGCCACGATGCCAAGGCACCTGCCCTCGACAACCACTGCTGCCCGCCTTACAACCCCAGCGAGACGATCTCGGAGTACGGCTTCCCCGCTACGCCCCTCCGGGTGCGGCGGCCGGCCCACCTCGTGAAGGACGATCAGGAGTATTTGGACAAGTACAAGGAGGCCGTGAGGAGGATGAAGAATCTGCCGGCAGACCACCCTTGGAACTACTACCAGCAGGCGAACGTCCACTGCCAGTACTGCAACTACGCCTACTACCAGCAAAATACCGACGACGTGCCCGTCCAGGTCCACTTCAGCTGGATCTTCCTCCCCTGGCACCGCTACTACCTCCACTTCTACGAGCGGATCCTCGGCAAGCTCATCGACGACGACACCTTCACCATCCCCTTCTGGAACTGGGACACCAAGGACGGCATGACGTTCCCCGCCATCTTCCACGAAGAGTCATCCCCGCTGTCTGACACGAAACGCGACCAACGCCACGTCAAGGACGGCAAGATCGTCGACCTCAAGTACGCCTACACCGAAAACCCTGCCTCCAACAGCGAGATCATTCGAGAGAACCTCTGCTTCATACAGAAGACGTTCAAGCACAGCCTGTCGCTGGCGGAGCTGTTCATGGGGGATCCCGTGCGCGCGGGGGAGAAGGAGATCCAGCAGGCTAACGGGCAGCTGGAAGCCATCCACAATGCGGTGCACATGTGGGTCGGAGAGCCGTGCGGATACAAGGAAAACATGGGCGACTTCTCTACCGCCGCCCGCGATTCTGTTTTCTTCAGCCACCACTCCAATGTCGACCGCTTGTGGGAAATCTACCGGAACCTCCGCGGTAACCGCATTGAGTTCGAAGACAACGACTGGTTGGACAGCACCTTCCTCTTCTACGACGAGAACGAGAAGCTCGTCAAAGTCAAGATGGGGGACTGCCTCAACCCGACCAAGCTTCGGTACACGTTCGAGCAAGTTCCTCTCCCATGGCTGGGCAAAATTAATTGCCAGAAGACGACAGAGACGAAGTCCAAGTCCACGACAGAGATGTCGCTGACGCGCGTGGGAGAATTCGGGACGACGCCCAAGGCGCTCGACGCGAGCAACCCGCTGCGGGTGATCGTGGCAAGGCCGAAAAAGAACCGCAAGAAGAAGGAGAAGCAAGAGAAGGTGGAGGTGCTTCAGATCAATGATATTAAGGTGACCACCAACGAGACAGCTCGCTTCGACGTCTACGTCACGGTTCCTTACGGTGACCTCGCCGGCCCGGACTACGGCGAGTTCGTGGGCAGCTACGTGAGGCTGGCGCATAGGATGAAGGGAAGCGAGGGGACCGAAGTGCAGGGCCCCAAGAAGAAGGGAAAGCTCAAGCTAGGTATTACGCCACTGCTCGAGGACATCGATGCTGAGGACGCCGACAAGTTGGTGGTCACCCTGGTTCTCCGCACCGGGAGCGTCACCGTGGGCGGAGTTTCCATCAATCTCCTGCAGACAGATTCTACCGCCGCCATCTAA*

**>MaPPO10-CDS**

ATGGAGGGCAAACGATGGTTGTCTCTTCTCCTCCTCGTGCTTGTTCTCGTGGGCATCTCCATGGATCTCCCGAGAGAAGCTCCGGCGGCTTCTTCTAATATCTTGAAGAGTTCATCTGCCAGAATACCAGTGAATCCTCAAGGCGGACAACAGAGAGATGGAAGCAAGAGCAGGGAGGACAAGGGCATTCCCCTCAAAGCAAACCTATCGGTTTGCCATGCTTCATTCTCGGACGCCGATCGTCCGGTCTACTGTTGCCCGGCCTGGAAAGACGCCGACCAAACCTTGCTCGACTTCGAGTTCCCGGATCCGTCGTCGCCGGTGCGTATCCGACGGCCTGCTCATCTCGTCGACGAGGAGTTCGTGGCCAAGTATGAGAGGGCGGTGGCCATCATGAAGCAGATCCCGCCTGACCATCCCCACAACTTTTGGCGCCAGGCCAACATGCACTGCCTCTACTGCACCGGCGCCTACGACCAGATGAACTCCTCTGCCCTCTTCAAGATCCACAGGTCATGGCTCTTCTTCCCCTGGCACCGAGCCTTCATCTATTTCCACGAGCGCATCCTCGGGAAGTTCATGGGAGACGACACCTTCGCGCTCCCCTACTGGAGCTGGGACACCCCCGAGGGCATGTGGTTCCCCGACATCTACCGGAAGGGAGCTCTGAATGAGACAGAGCGCGACGCCATTCACCTACGGGAGGCCGCCGTCGATGACTTCGACTACGTGGATCATGACCTAGACAGCGACGTGCAGATCGCCGACAACCTCGCGTTCATGTACCACCAGATGATCTCGGGAGCGAAGAAGACCGAGCTGTTCATGGGTTGCAAGCTGCGGTCCGGCGTCGAGGGGTGGTGTGATGGGCCCGGGACGATCGAAGCAGCACCTCACAACACGTTGCACAGCTGGGTGGGGAACAGGTACAACCCCGAAAGAGAGAACATGGGGGCGTTCTATTCCGCCGCGCGAGACGAAGTGTTCTTCGCGCACCACTCCAACATCGACCGCATGTGGACGGTGTGGAAGAAGCTGCACGGCGACAAGCCGGAGTTCGTCGACCAGGAGTGGCTCGAGTCTGAATTCACCTTCTACGACGAGAATGTGCGCCTGCGCAGGATCAAGGTGCGCGACGTGTTGAACATAGACAAACTCAGGTACCGGTACGAAGACATCGACATGCCATGGCTCGCTGCACGTCCCAAGCCTTCCGTTCACCCTAAGATCGCGCGCGACATATTGAAGAAGCGTAATGGCGAAGGCGTACTGAGAATGCCCGGCGAAACGGATCGTTCACAACTCTCCGAAGATGGTAGCTGGACACTGGACAAGAGCATCACCGTGAGGGTTGACAGGCCAAGGATCAACAGGACAGGGCAAGAAAAAGAGGAAGAAGAGGAGATCTTATTGGTCTACGGAATCGATACTAAGAGAAGCAGATTTGTCAAATTCGATGTGTTCATCAACGTCGTCGACGAAACCGTGCTGAGCCCAAAGTCGAGGGAGTTCGCAGGGACCTTCGTAAATCTCCACCACGTCTCGAGGACGAAAAGCCATGAGGATGGCGGCGTGGGTTCGAAGATGAAAAGCCACCTTAAGCTCGGTATATCGGAACTTTTGGAAGACCTCGAGGCAGACGAAGATGACAGCATCTGGGTGACACTGGTGCCAAGAGGCGGCACGGGGGTCAACACCACCGTAGACGGCGTCCGGATCGACTACATGAAGTAG*
